# Limblab: pipeline for 3D analysis and visualisation of limb bud gene expression

**DOI:** 10.1186/s12859-025-06264-4

**Published:** 2026-01-12

**Authors:** Laura Aviñó-Esteban, Heura Cardona-Blaya, Marco Musy, Antoni Matyjaszkiewicz, James Sharpe, Giovanni Dalmasso

**Affiliations:** 1 European Molecular Biology Laboratory (EMBL-Barcelona), 08003 Barcelona, Spain; 2 Universitat Politècnica de Catalunya - BarcelonaTech (UPC), 08034 Barcelona, España; 3 Barcelona Collaboratorium for Modelling and Predictive Biology, 08005 Barcelona, Spain; 4 Institució Catalana de Recerca i Estudis Avançats (ICREA), 08010 Barcelona, Spain; 5 Department of Mathematics and Data Analysis, IQS, Universitat Ramon Llull, 08017 Barcelona, Spain

**Keywords:** 3D visualization, Limb development, Gene expression analysis, Bioinformatics pipeline

## Abstract

**Background:**

Although some aspects of limb development can be treated as a 2D problem, a true understanding of the morphogenesis and patterning requires 3D analysis. Since the data on gene expression patterns are largely static 3D image stacks, a major challenge is an efficient pipeline for staging each data-set, and then aligning and warping the data into a standard atlas for convenient visualisation.

**Results:**

We present a novel bioinformatic pipeline tailored for 3D visualization and analysis of developing limb buds. The pipeline integrates key steps such as data acquisition, volume cleaning, surface extraction, staging, alignment, and advanced visualization techniques. Its modular design allows researchers to customize workflows while maintaining compatibility with tools such as Fiji and Vedo. The pipeline can be accessed at https://github.com/LauAvinyo/limblab.

**Conclusions:**

The pipeline advances 3D gene expression analysis in limb development by integrating flexible tools for staging, alignment, and visualization. It is user-friendly, scalable to other samples, and optimized for research needs. Future updates will enhance customization and expand applicability to other species and developmental biology fields.

## Background

The spatial organization of cells and their interactions is crucial to understand various biological processes, including development, differentiation, and disease progression [1]. Understanding the development of tissues and organs often requires analysis of complex three-dimensional (3D) structures, as two-dimensional (2D) data do not capture all the key architectural and geometric features [2, 3]. For example, gene expression patterns, when captured in 2D, may fail to accurately represent the spatial relationships between genes and other molecular entities [4]. This issue is particularly evident in the development of the mouse limb, where spatial context is crucial [5]. Although the limb can sometimes be simplified to a 2D problem, such reductions can create false impressions of gene expression patterns and their interactions.

Advances in imaging technologies and computational methods have facilitated the collection of large amounts of 3D data. High-resolution imaging techniques, such as confocal microscopy [6], Optical Projection Tomography (OPT) [7], and light sheet fluorescence microscopy, allow researchers to acquire detailed 3D datasets of tissues and organs [7–10]. Structural imaging modalities, such as Magnetic Resonance Imaging (MRI) [11] provide anatomical context [12]. This high amount of data provides an invaluable resource for accurately mapping gene expression and understanding the dynamics of developing limb buds and other tissues.

In species such as Zebrafish and Drosophila, which develop externally, it is possible to use time-lapse imaging to effectively make movies of the entire developmental process in vivo and in real-time [13, 14]. In contrast, this approach remains challenging for embryos that develop internally, particularly after the gastrulation stage [15]. In vitro culture systems face significant limitations in recapitulating complete mouse embryogenesis. For instance, it is difficult to replicate the later stages of mouse development beyond embryonic day 10.5 (E10.5) in a set-up that allows in vitro time-lapse imaging [16, 17].

Hence, in internally developing organisms, it is often necessary to collect and fix the embryos and stage the limbs post-collection. Currently, several approaches exist for embryo staging, each with distinct advantages and constraints. The most commonly employed method relies on chronological timing from fertilization; however, this approach lacks precision due to natural developmental variability between embryos and litters [18]. Morphological landmark-based methods offer improved accuracy but present practical limitations: somite counting, while highly reliable, requires access to the complete embryo; other organ-specific or anatomical milestones similarly necessitate full embryonic specimens and may not be consistently visible across all developmental stages. All of this makes accurate staging essential for meaningful comparisons and understanding the temporal dynamics of gene expression during limb development. This requires robust methods for staging limb buds independently to ensure that comparisons are made between equivalent developmental stages [19, 20].

To address this challenge, Musy et al. [20] developed a geometric morphometric approach that overcomes many limitations of traditional staging systems. Their embryonic Mouse Ontogenetic Staging System (eMOSS) quantifies 2D limb bud shape changes between approximately embryonic days E10 and E13. The system constructs a continuous “reference trajectory” of curvature profiles derived from splines of dorsal-view limb images, against which new limb images are compared through alignment and scaling procedures. This methodology yields age estimates with remarkable precision–approximately ± 2 h uncertainty for the size-dependent version, or ± 3 h for the size-independent variant. Importantly, the method demonstrates robustness to variations in orientation (up to $\pm \,15^{\circ }$) and user input (standard deviation  45 min across novice users), while remaining resilient to tissue processing artifacts such as fixation and dehydration when employing the size-independent algorithm.

There are several general tools for 3D data analysis available. For instance, Fiji, Napari, Imaris, and Paraview, offer powerful general-purpose 3D image analysis and visualization capabilities [21–23]. While these platforms provide excellent foundational tools for 3D visualization and analysis, they present significant limitations when applied to limb development research. First, they lack specialized functionality for developmental stages, such as the staging systems that can accurately classify embryonic limb buds. Second, they do not provide integrated workflows for limb-to-reference alignment protocols that are needed for comparative gene expression analysis across different samples and developmental stages.

Users could theoretically circumvent these limitations by combining multiple software packages to achieve a complete analysis. However, this approach introduces practical challenges that make it impractical for most researchers. Such multi-platform workflows require users to master diverse computational environments. For instance, one might need to use Fiji [21] for initial image processing, R or Python for statistical analysis, specialized mesh processing software like MeshLab [24] for 3D operations, and separate visualization tools. They would also require the implementation of custom integration with a staging system and anatomically accurate reference limbs. This approach demands technical expertise across different programming languages and interfaces, creating a steep learning curve for non-expert users. Beyond the expertise barrier, these multi-platform solutions require considerable time investment to develop custom scripts for data transfer between platforms, often involving multiple file format conversions and manual parameter adjustments at each step. This multi-platform approach introduces potential compatibility issues and version dependencies that can break workflows when software updates occur, requiring constant maintenance and troubleshooting.

Consequently, there is a clear need for a specialized computational framework that offers a self-contained, integrated workflow for comprehensive analysis of 3D gene expression data in limb development. Here, we present LimbLab, an open-source tool that provides a unified environment for all necessary operations, from raw data import through 3D visualization, developmental staging, and mesh alignment, within a single interface designed specifically for limb development studies. LimbLab integrates eMOSS and standardized anatomical reference models. Finally, it runs on top of VTK [25], hence, providing robust 3D rendering capabilities for state-of-the-art visualization and customization.

## Implementation

In this paper, we present a novel bioinformatics pipeline designed specifically for 3D visualization and analysis of developing limb buds. This pipeline addresses critical challenges in the field, such as the individual staging of limb buds, aligning or morphing to a canonical shape, and general tasks such as data processing and visualization of different volume channels. The different steps of the pipeline are:

- Data acquisition: Capturing high-resolution 3D images of limb buds, and gene expression patterns using imaging techniques such as HCR [26], OPT [7] and light-sheet microscopy [8].
- Data cleaning: Removal of noise and artifacts from the raw data to ensure high-quality input for the next steps.
- Limb staging: Independent staging of limb buds providing the morphological stage [20] of the considered limb.
- Transformation: Alignment or morphing of the limb buds to a canonical shape to facilitate consistent comparisons between samples.
- Visualization: Generating detailed 3D rendering for in-depth inspection and interpretation of gene expression patterns.

**Fig. 1:**
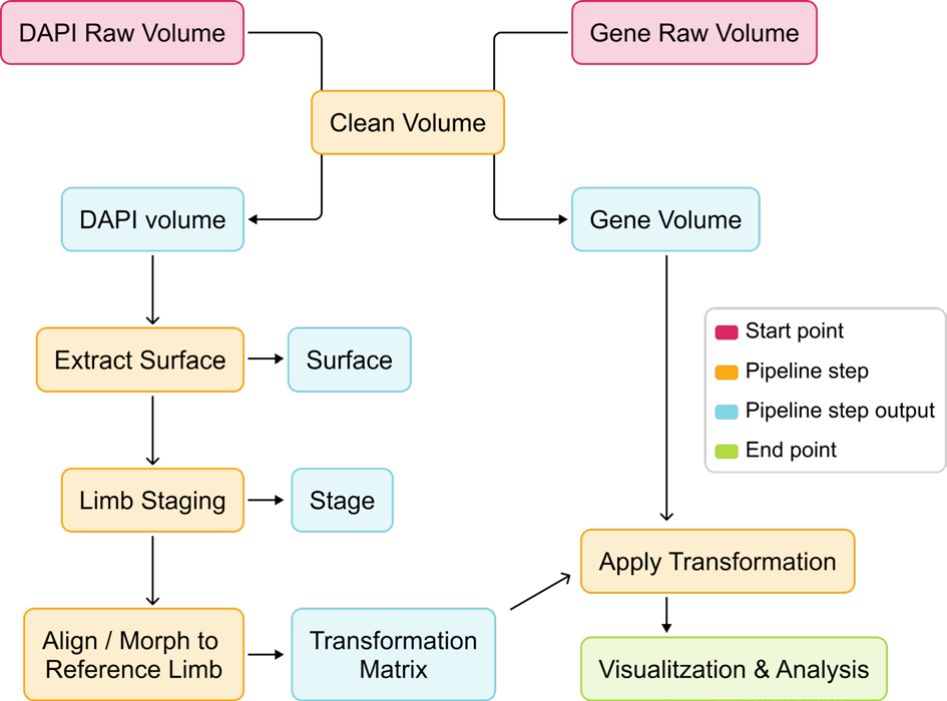
Bioinformatics pipeline for 3D limb bud analysis. Schematic representation of the pipeline steps: data acquisition (red), data cleaning and transformation (orange), visualization (green), outputs (blue)

A schematic representation of these steps is shown in Fig. 1, providing an overview of the process and how each component integrates to facilitate a comprehensive analysis.

We implemented the command-line interface (CLI) using Python [27] with Typer [28]. The CLI integrates with our 3D visualization and plotting tools built on Vedo, for the rendering of volumetric data and interactive analysis [29] (see Methods). We implemented an API for eMOSS [20] and deployed it publicly at [https://limbstaging.embl.es/api]. The API was developed using Python [27] with the FastAPI [30] framework.

## Results

The implementation of our bioinformatics pipeline provides a robust framework for analysing 3D gene expression data in developing limb buds. This pipeline integrates several crucial steps to facilitate the accurate and efficient analysis of complex volumetric datasets. In this section, we detail each stage of the pipeline. We demonstrate how the pipeline effectively preprocesses raw imaging data, extracts critical anatomical surfaces, and accurately stages and aligns limb buds. In addition, we present advanced visualization tools that allow for an intuitive and detailed exploration of gene expression patterns in 3D.

### Data pre-preprocessing

The primary requirement of the pipeline is a channel with nuclei labeling, such as DAPI, or any other structural image of the limb, from which to extract the organ’s outer surface, which is needed for accurate limb staging and alignment to a reference limb. The pipeline assumes that all channels within the same volume are pre-aligned, a condition typically already met by most multi-channel microscopy and mesoscopic techniques.

Before cleaning the volumes, the user is prompted to create a folder, referred to as the *experiment folder*, where all intermediate steps and results will be saved. During this process, the user will also specify whether the limb is *right* or *left*, *hindlimb* or *forelimb*, as well as the microscope voxel dimensions. Within the experiment folder, a file named pipeline.log will be generated. This file will automatically store all values specified by the user (e.g. clip values used during volume cleaning) and subsequently all automatically assigned values (e.g. the morphological stage), ensuring full reproducibility of the analysis workflow.

**Fig. 2:**
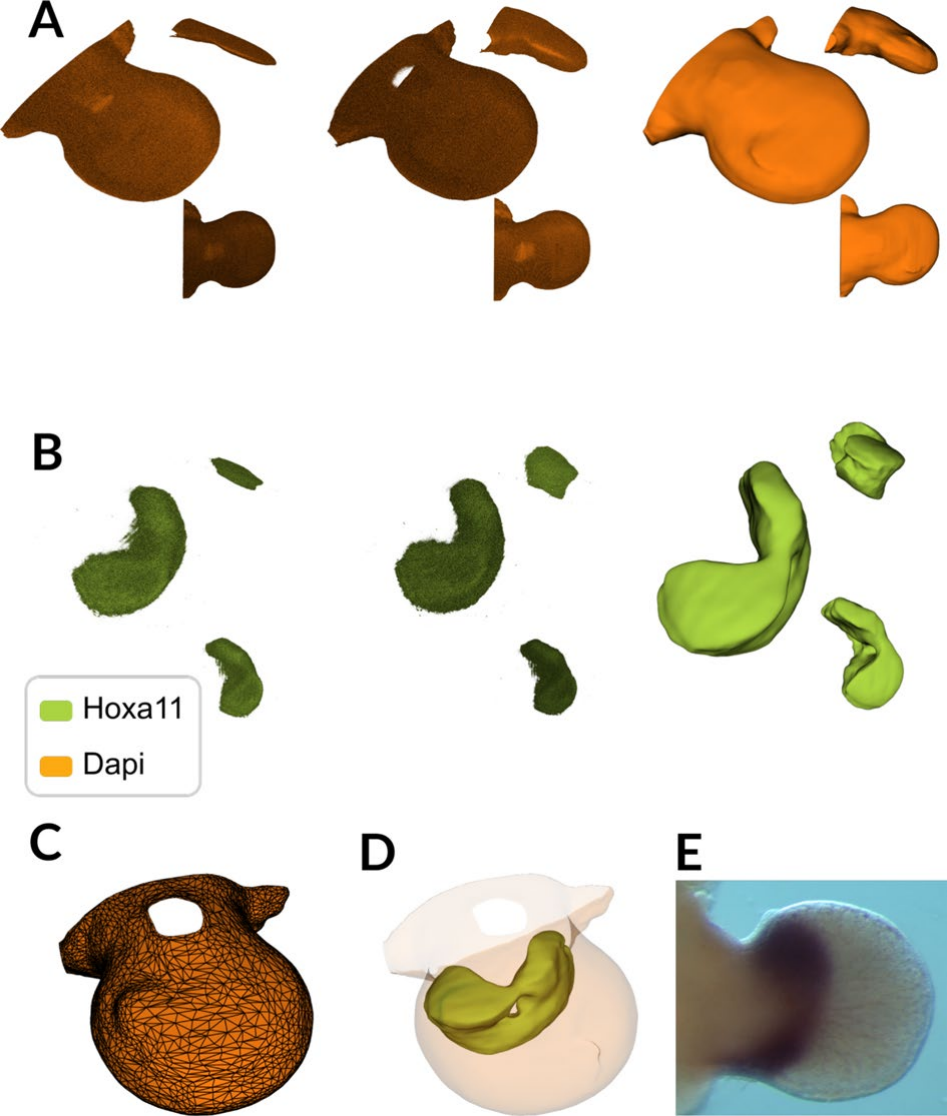
Data cleaning and surface extraction **A** Representation of the cleaning steps of a limb bud volume using the DAPI channel. First column shows the row data, second column shows the data after correcting for the voxel spacing, third column shows the volume after the noise reduction. See Movie 1. **B** Same process show in (A) but for the channel of the *Hoxa11* gene. **C** Representation of the extracted surface of the limb. See Movie 2. **D** Visualisation of the limb surface extracted from the from DAPI volume (light orange) and an isosurface of *Hoxa11* gene volume (green). **E** Whole-mount in situ hybridization image showing the expression pattern of the *Hoxa11* gene in a developing mouse limb bud

The user can then begin the volume cleaning process using the command clean-volume. The script first adjusts for the microscope spacing as specified in the previous step to ensure accurate spatial measurements, then prompts the user to select bottom and top isovalues through an interactive browser interface to eliminate intensity outliers and preserve only the tissue-relevant signal range. These thresholds are automatically logged for reproducibility. For data acquired from left limbs, the pipeline applies mirroring to enable comparison with the right-sided reference system. The script subsequently applies a series of filtering operations: Gaussian smoothing with a default sigma of (6, 6, 6) voxels to reduce high-frequency imaging noise while preserving morphological features, followed by a low-pass filter with a default cutoff frequency of 0.05 to remove residual acquisition artifacts and enhance surface coherence (Fig. 2A, B, and Movie 1). These default values were selected empirically through visual validation across different HCR volumes to optimize the balance between noise reduction and feature preservation; nevertheless, the user is encouraged to fine-tune the values based on their data and microscope setup. The volume can then be resized to standardized dimensions to optimize computational efficiency for downstream surface extraction and alignment operations. Finally, the refined data are saved as a vti format file in the experiment folder, ensuring that the volume is ready and easily accessed for further analysis.

### Surface extraction

To optimize staging and alignment, the surface of the organ is extracted as a polygonal mesh, which is computationally more efficient than aligning or morphing raw volume data. This organ surface delimitation is typically performed using the channels from nuclear staining dyes such as DAPI, which binds strongly to adenine-thymine-rich regions in DNA, or DRAQ5, a far-red nuclear stain that labels dsDNA in live cells. These fluorescent nuclear markers delineate tissue boundaries by visualizing cell nuclei distribution rather than specific gene expression patterns, providing anatomical information essential for accurate surface definition. However, LimbLab’s capabilities extend beyond nuclear staining to accommodate any scalar volumetric dataset–once preprocessed through our pipeline or external software, diverse structural imaging data can be analysed using the same surface-based computational tools.

Users can select the isosurface for the outer limb surface either programmatically or through a user interface (UI), with the option for full automation through the pipeline’s ability to identify and apply the most frequent isovalue for extraction. The selected isovalue is stored in the pipeline.log file for reproducibility. Once the isovalue is established, the corresponding surface is extracted and processed through a series of optimization steps. The script first isolates the largest connected component of the surface mesh to remove residual tissue fragments and imaging artifacts. To reduce computational complexity while preserving both volumetric and geometric fidelity, it then applies VTK’s vtkQuadricDecimation filter, retaining, by default, just 0.5% of the original triangles (target reduction=0.995). This default value provides a good balance between computational efficiency and geometric accuracy for our data, though users can adjust this parameter to suit their specific requirements and tolerance for detail preservation. We use this filter due to its built-in volume-preservation option, that augments the standard quadric error metric with a penalty on deviations in triangle normals and local volume change, ensuring that only the most geometrically significant vertices are retained and that surface orientation is faithfully maintained. The result is a simplified mesh that remains quantitatively and visually congruent with the original geometry (Fig. 2C, D and Movie 2).

### Staging of the limb

**Fig. 3:**
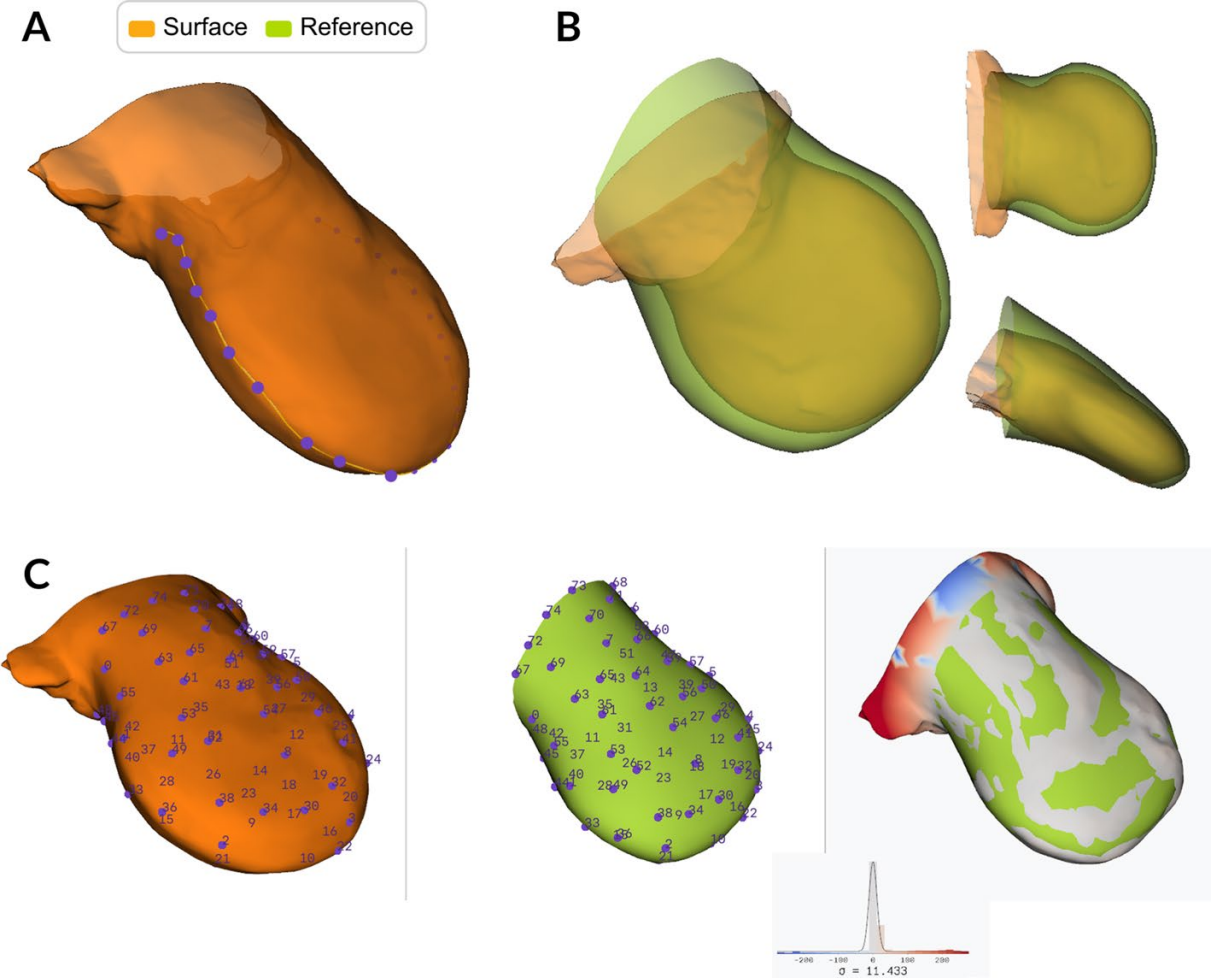
Staging, aligning and morphing of the limb **A** Visualisation of the interactive plotter for the manually selection of the Apical Ectodermal Ridge (AER). The user must click the limb surface to select the points (purple) that determine the spline of the AER curve (yellow). See Movie 3. **B** Interactive alignment of the limb (orange) to the reference (green) using linear transformation. The user can manually move and rotate the limb to align it with the reference. See Movie 4. **C** Interactive non-linear transformation tool designed to morph a limb to match a reference. Users select pairs of corresponding points (purple) between the limb and the reference. Once a sufficient number of pairs are selected, the tool can automatically generate additional point pairs based on the closest distances. These points collectively define the non-linear transformation used for morphing. Upon completion, the third column displays the distance between the two meshes, providing a visual representation of the alignment accuracy. See Movie 5

The user can stage - that is, determine the developmental age of a limb—using the command stage. This process involves the manual identification of the Apical Ectodermal Ridge (AER) using the interactive plotter (Movie 3). Once the selection is made, the system sends an API call to the staging server [20]. The server returns the assigned limb morphological stage, along with the accuracy of the staging and a graphic summary of the process. The morphological stage is saved for use in the subsequent steps of the pipeline (Fig. 3A and Movie 3).

### Aligning/morphing of the limb

With the align command, the user can align the data to a reference limb [31, 32]. This alignment can be performed using one of two transformation approaches, each suited to different requirements and data characteristics. The first method (command: align) employs a linear transformation that allows the user to interactively rotate and scale the limb while preserving the original shape of the surface (Fig. 3B and Movie 4). This approach is optimal when the sample and reference exhibit similar overall morphology and only require adjustment for orientation, making it particularly suitable where shape preservation is critical. The user uses the GUI to align the limb to the reference until satisfied with the alignment.

The second method (command: align –morph) utilizes a non-linear transformation approach for cases the user needs the limb and the reference to match as precisely as possible and does not require having shape preservation in the data. In this method, the user establishes correspondence by selecting matching landmark points on both surfaces, beginning with key anatomical features such as the apical ectodermal ridge, digital rays, or limb bud boundaries. Initially, the user selects a few strategic points to achieve a rough alignment, after which the script automatically refines the alignment through iterative closest point matching between the surfaces. The user maintains control over alignment precision by iteratively adding or modifying landmarks and evaluating the resulting surface-to-surface distance maps until satisfactory correspondence is achieved. This approach accommodates shape variations and is particularly valuable when comparing gene expression patterns from different samples (Fig. 3C and Movie 5).

Although the alignment process operates on the extracted surface, the resulting transformation matrix can be applied to the entire volume, ensuring spatial consistency across all data channels. For both methods, the transformation parameters are stored for subsequent application to gene expression pattern volumes, enabling reproducible registration workflows.

### Analysis and visualization

The LimbLab package provides a comprehensive suite of visualization tools designed to accommodate various research objectives, allowing precise exploration and analysis of 3D gene expression data. All of these visualization capabilities are built on top of the Vedo library, a versatile and powerful tool for scientific analysis and 3D visualization [29]. In addition, this package is designed with flexibility in mind, making it easy to add new visualization methods as research needs evolve. All visualizations are shown in Movie 6.

#### Slices

**Fig. 4:**
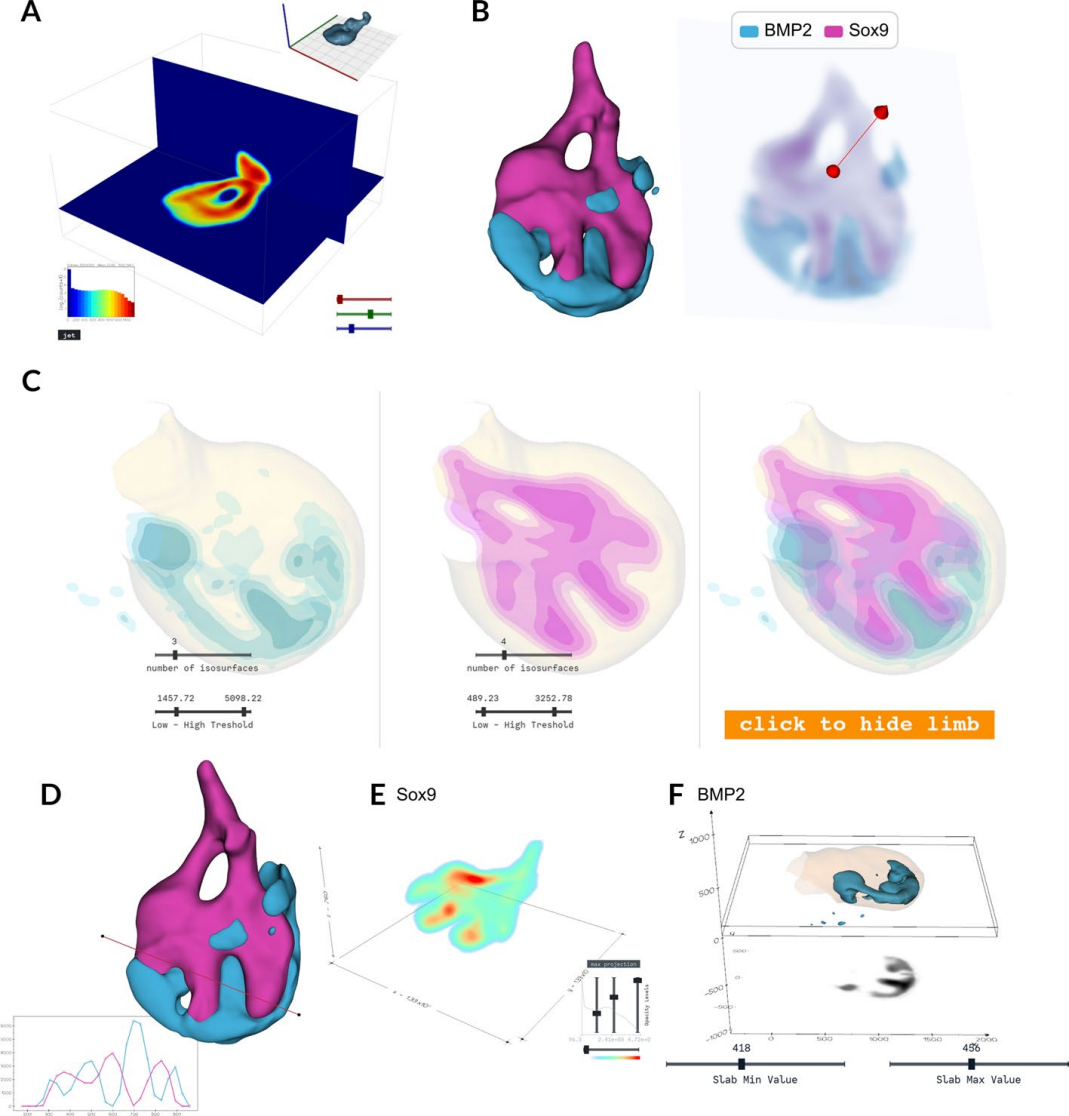
Analysis and visualization See Movie 6. **A** Slicing of the 3D expression of the *Sox9* gene (top right) along the x, y, and z planes. The colour represent the intensity values of gene expression, which are also depicted in a histogram (bottom left). The user can change colormaps. Sliders allow interactive movement of the x, y, and z planes (bottom right). **B** Visualization of the genes *BMP2* and *Sox9* (left) and an arbitrary slice of these two channels (right). **C** Isosurface visualization of the volumetric data of a limb (beige) and the expression of the genes *BMP2* (blue) and *Sox9* (pink). Various sliders enable interactive changes to the number of isosurfaces and the lower and upper threshold values (bottom). A button allows hiding and showing the limb isosurface (bottom right). **D** Probing two volumes of gene expression, *BMP2* (blue) and *Sox9* (pink), with a line, alongside a plot of their respective intensity values. **E** Raycast visualization of the *Sox9* gene, with a colour scheme representing gene expression levels. Sliders adjust opacity of gene expression levels, and the colour mapping (bottom right). **F** Representation of a slab (black) extracted from *BMP2* volumetric gene expression (blue). Sliders control the slab’s minimum and maximum values (bottom)

Displaying slices along the x, y, and z planes allows the researcher to examine cross-sections of 3D data at various depths, providing a better view of internal structures and spatial distributions. The detailed 2D slices along these planes help reveal gene expression across different layers of the biological sample. Interactive sliders enable users to dynamically adjust slice positions, making it easier to focus on specific regions of interest. An example of the visualization of the *BMP2* gene using this method is shown in Fig. 4A. Additionally, it is possible to visualize the pairing of different genes and extract arbitrary slices of multiple channels. This is shown using the genes *BMP2* and *Sox9* in Fig. 4B.

#### Isosurface visualization

Isosurface visualization represents three-dimensional volumetric data by plotting surfaces through regions of constant value. This method effectively visualizes gene expression patterns by allowing users to delineate areas where expression meets specific thresholds. By generating isosurfaces at different values, users can clearly identify and analyse the spatial distribution of gene activity within limbs or organs, facilitating a more intuitive understanding of complex 3D biological data. A key advantage of isosurface visualization is its ability to display multiple gene expression patterns simultaneously in the same 3D space. The overlay of isosurfaces for different genes enables direct observation of spatial relationships, colocalization, and interaction patterns between genes (Fig. 4C).

#### Probe

Probing a gene expression volume with a line and plotting intensity values provides another way of analysing gene activity in 3D. This involves drawing a line through the volume to examine variations in gene expression, allowing researchers to gain insights into spatial patterns and specific regions of interest. Furthermore, the same line can be applied across different channels, enabling comparative analysis across channels and datasets (Fig. 4D).

#### Raycast visualisation

Raycasting is a powerful technique for visualizing 3D volumetric data, such as gene expression patterns, obtained by simulating rays passing through the data to reveal internal structures. LimbLab also includes controls for adjusting the visualization, such as sliders for opacity and colour mapping, allowing users to fine-tune how gene expression levels are represented in 3D space. The opacity sliders adjust the transparency of different volume parts, enabling users to focus on areas of interest by making other regions more transparent. Colour mapping further enhances visualization by applying various colour schemes to different gene expression levels, highlighting specific gene activity thresholds (Fig. 4E).

#### 2D Projection slab

Extracting a slab from a volumetric dataset simplifies gene expression analysis by converting a 3D volume to a 2D thick slice. This technique combines slices along an axis to create a single image, making it easier to examine gene expression within a specific spatial range. Focusing on a slab helps identify patterns and variations in gene activity, which is particularly useful for studying specific developmental stages or areas with significant changes. Projecting the slab using mean projections highlights different aspects of gene expression: the mean projection shows the average levels, while the maximum projection emphasizes the highest activity. The metadata stored with the slab object, including the range, axis, and operation of the slab, supports a precise and reproducible analysis (Fig. 4F). To ensure that the slab is perpendicular to the limb, the gene expression volumes and the surface are rotated to correct for variations in the angle of limb protrusion relative to the embryo’s flank [31].

### Hoxa11 case study

During vertebrate limb development, the growing limb bud is patterned along three distinct axes, with the proximal-distal (PD) axis representing the "horizontal" dimension that extends from the body wall toward the limb tip. The PD axis establishes the sequential development of limb segments from stylopod (upper arm/thigh) to zeugopod (forearm/lower leg) to autopod (hand/foot), with proximal identity maintained by retinoic acid (RA) signalling that activates *MEIS1* and *MEIS2* transcription factors, while distal identity is promoted by *FGF* signalling from the apical ectodermal ridge. This axis is further refined by the nested expression of *Hox* genes, where *Hoxa11* and *Hoxd11* specify zeugopod (radius/ulna) identity and coordinate chondrocyte differentiation, while *Hoxa13* and *Hoxd13* control autopod (hand/foot) development and digit formation  [33].

**Fig. 5:**
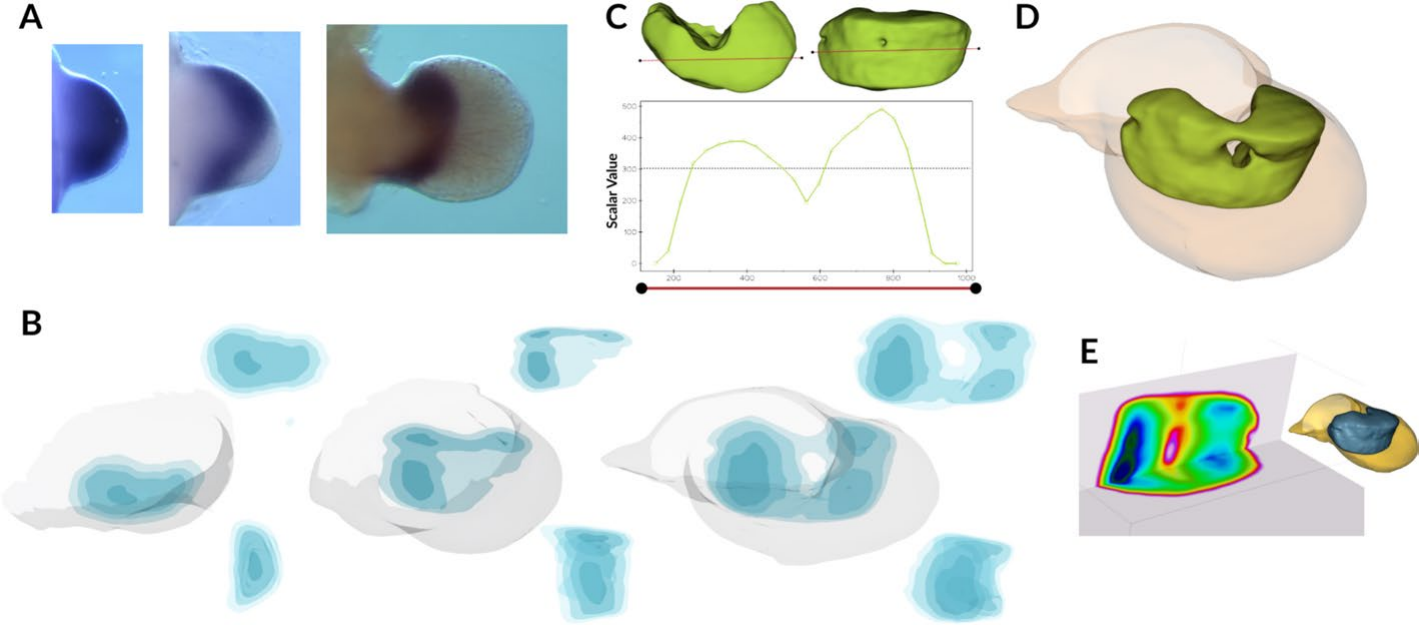
*Hoxa11* expression reveals non-uniform spatial distribution during limb development. **A** 2D time-course showing *Hoxa11* expression as its characteristic stripe pattern. **B** 3D time-course analysis of *Hoxa11* expression in developing limbs (E10:05, 11:09 and 11:19). Top: diagonal view; bottom: lateral view. Expression transitions from uniform stripe to donut-like pattern at later time points. **C** Expression profile along the red line in top *Hoxa11* surface renders, showing scalar values representing *Hoxa11* levels. **D** Isosurface rendering at scalar value 300 (dotted line in C) demonstrating the donut-shaped expression domain. **E** Vertical and horizontal cross-sections of the same volume illustrating spatial heterogeneity of *Hoxa11* expression

To test the practical utility of our 3D visualization pipeline in revealing biologically meaningful spatial patterns that are obscured by conventional 2D analysis, we explored the expression domain of *Hoxa11* on days 10, 11, and 12 after conception. When examined through traditional WISH 2D images, *Hoxa11* expression consistently appeared as a stripe pattern as seen in Fig. 5A , which would typically be interpreted as a linear or banded gene expression domain. However, application of limblab isosurface rendering (Fig. 5B), probe quantification (Fig. 5C), and slices visualization (Fig. 5E) across three *Hoxa11* volumetric datasets from around the same ages revealed that these apparent ’stripes’ are a more heterogeneous pattern. As shown in figure, at the first time point, there is a horizontal gradient. At the second time point, the expression resolves into a bilobed pattern, with two distinct expression maxima and a reduced signal in the centre, indicating an emerging ring-like structure. Finally, for the third time point, the expression shows the ring-like expression domain. The expression pattern is like a donut (Fig. 5D), with a hole in the middle, that cannot be detected by normal 2D analysis. This case study demonstrates that a 3D visualization approach can fundamentally alter biological interpretation by revealing the true spatial organization of gene expression patterns that are systematically misrepresented when analysed solely through 2D methods.

## Conclusion and discussion

The bioinformatics pipeline presented in this work represents a significant advance in the field of 3D gene expression analysis, particularly in the context of limb development [5]. By integrating a comprehensive suite of tools for data acquisition, volume cleaning, surface extraction, staging, alignment, and visualization, this pipeline addresses the complex challenges inherent in analysing 3D limb bud data [1–4]. Its modular design ensures flexibility, allowing users to tailor the workflow to their specific research needs while maintaining compatibility with widely used tools (e.g., Fiji [21] and Vedo [34]).

While scientific visualization tools, like Fiji and Napari, are excellent general-purpose tools for image analysis and offer robust functionality for 2D image processing and stack visualization, they present limitations when it comes to 3D rendering and interaction. Although these platforms can display volumetric data through stack-based visualizations, working with 3D objects in these environments often feels clunky and restrictive. Our pipeline addresses these limitations by using VTK’s robust 3D visualization framework through the Vedo library, which provides native support for mesh rendering, intuitive interactive navigation, and real-time geometric transformations. This approach enables seamless manipulation of 3D anatomical structures with responsive performance and visual quality.

One of the key strengths of this pipeline lies in its specialization and integration of cutting-edge limb developmental biology methodologies. While general tools provide broad applicability, they often lack the convenience and specificity required for niche applications. Our pipeline, on the other hand, is purpose-built to tackle the specific challenges of limb development studies by incorporating the embryonic Mouse Ontogenetic Staging System (eMOSS) for accurate temporal classification and the specialized anatomical reference system developed by Dalmasso et al. [31] for precise spatial alignment, while still offering smooth visualization tools.

The pipeline’s flexibility extends beyond traditional gene expression analysis methods to accommodate the evolving landscape of spatially resolved transcriptomics technologies. While initially designed for data from HCR, OPT, and light-sheet microscopy, LimbLab is inherently compatible with high-throughput spatially resolved transcriptomics methods. The key requirement is that the input data consists of spatial coordinates with associated scalar values, which is the standard output format after barcode decoding and gene assignment in multiplexed approaches. This data format agnosticism allows the pipeline to handle both single-molecule resolution data (where spatial coordinates of individual transcripts are stored) and cell-resolution data (where transcripts are segmented into cells with centroid coordinates), providing researchers with the flexibility to work across different experimental methodologies and data acquisition techniques.

A user-friendly interface and robust documentation make the pipeline accessible to researchers with varying levels of expertise, thereby lowering the barrier entry point for complex 3D data analysis. Although the current implementation is optimized for mouse limb buds, the surface extraction and visualization capabilities of the pipeline are applicable to a wide range of biological samples, offering the potential for a wider impact in the field of developmental biology. Moreover, the visualization parameters, such as colour schemes and UI positions, are only defined once in the code; hence, it streamlines the creation of consistent publication-ready figures, saving valuable time and effort for researchers.

To ensure that our pipeline evolves in line with the needs of the limb development research community, we actively seek feedback and new requirements from researchers. This engagement will help us tailor future updates and improvements. Upcoming releases will feature new visualizations and additional customization options based on this feedback. Moreover, we plan to explore the integration of tools for analysing other model species, such as chick and axolotls, which will extend the utility of LimbLab to other fields like evolutionary development and regeneration. Future developments could include the integration of additional staging systems or reference datasets, as well as enhancements to the current visualization techniques to accommodate even more complex biological structures. As the field of developmental biology continues to evolve, tools like this pipeline will be essential in driving forward our understanding of the intricate processes that govern development.

## Materials and methods

### Materials

#### Embryo collection

C52BL/6J mouse females were bred and humanely euthanised at different times during gestation to collect embryos at E10.5, E11.0, E11.5 and E12.0. Embryos were dissected and immediately fixed with 4% PFA overnight at 4 °C on a shaker. After that, embryos were washed few times with 0.1% PBT and gradually dehydrated with 25%, 50%, 75% and 100% Methanol dilutions in PBT and stored at −20 °C. The euthanasia of the pregnant females was done by cervical dislocation or using carbon dioxide just before isolating the uterus with embryos. When the late-stage embryos from E12.0 or later were required, the embryos were also quickly euthanised by decapitation just after isolation.

### Methods

#### HCRs

In order to perform the HCR, we followed the protocol exactly as described in [35]. In summary, HCR protocol consists of several steps: fixation, photochemical bleaching, detection, and amplification of HCR™RNA-FISH, clearing, and sample preparation.

#### Sample preparation

After clearing samples with Fructose-Glycerol solution for a couple of days, samples were embedded in 1% low melting agarose in 10 mM Tris pH 7.4. The agarose gel containing the sample was then cut into octagonal prism shapes of approximately 1 cm^3^ and placed on a 24 well plate with fructose-glycerol clearing solution in order to clear the agarose. This is incubated for a minimum of 24 h at room temperature protected from light.

#### Data acquisition

Light sheet imaging was performed using MuViSPIM from Luxendo. Dual side illumination was performed with the illumination objectives Nikon CFI Plan Apo Lambda 4$\times$ and collar was adjusted to 1.47, matching the refractive index from Glycerol-Fructose clearing solution. Fluorescence was then captured with immersion 10X Refractive Index adjustment 1.33$-$1.51 0.5NA glyc WD 5.5 objective. The block of agarose containing the sample was attached to a 3D printed holder and afterwards was immersed in the cuvette containing Fructose-Glycerol clearing solution.

#### Use of artificial intelligence tools

We used ChatGPT to aid in drafting and refining this manuscript.

### Software architecture and implementation

The software architecture is built around a command-line interface implemented using the typer library, providing a modular and extensible framework for processing 3D limb data. The core computational engine uses the vedo library, which provides a Python interface to the Visualization Toolkit (VTK) for advanced 3D data manipulation and rendering.

#### Core dependencies and technical stack

The software utilizes several key Python libraries: numpy and scipy for numerical computations, requests for API communication with remote staging services, and matplotlib for color interpolation and vedo data visualization. The underlying VTK framework provides robust implementations of computer graphics algorithms for isosurface extraction, mesh decimation algorithms, and spatial transformation operations.

#### Data management and reproducibility

The software implements a project-based data management system where each experiment is contained within a dedicated directory structure. A central configuration file (pipeline.log) maintains a key-value mapping of all pipeline parameters including file paths, processing parameters (e.g. voxel spacing, filter coefficients), and transformation matrices. This design ensures complete reproducibility of all processing steps and provides a clear audit trail for scientific validation.

### Technical implementation of the data processing pipeline

#### Volume Pre-processing and signal enhancement

Raw 3D image data, typically acquired as TIFF stacks from confocal or light-sheet microscopy, undergo a comprehensive pre-processing workflow. The processing pipeline begins with voxel spacing calibration, where physical dimensions are applied to the volumetric data based on microscope acquisition parameters (default: $0.65 \times 0.65 \times 2.0$ $\upmu$m^3^).

The pre-processing workflow consists of three main steps:

- *Intensity thresholding* An adaptive intensity segmentation approach is employed using an interactive IsosurfaceBrowser interface. Users define lower and upper intensity thresholds ($v_0$, $v_1$) to isolate the tissue of interest from background noise. The volume is then mapped using a colour transfer function: $V'(x,y,z) = \text {cmap}(V(x,y,z), v_{\text {min}}=v_0, v_{\text {max}}=v_1)$ where values below $v_0$ are set to 0 and values above $v_1$ are clamped to $v_1$.
- *Spatial normalization* All volumes can be resampled to a standardized any grid using trilinear interpolation to ensure consistent spatial resolution across samples. For bilateral symmetry analysis, left limbs undergo mirror transformation about the sagittal plane.
- *Noise reduction* A two-stage filtering approach is applied:
  - Gaussian smoothing with a default $\sigma = (6, 6, 6)$ voxels to reduce high-frequency noise
  - Frequency domain filtering with a default high-frequency cutoff at 0.05 cycles/voxel to eliminate imaging artifacts while preserving biological structures
  The processed volumes are saved in VTI format for efficient storage and subsequent processing.

#### Surface extraction using marching cubes algorithm

Three-dimensional surface meshes are extracted from the pre-processed volumes using VTK’s implementation of the marching cubes algorithm. The surface extraction process involves:

- *Isovalue selection* Three strategies are available:
  - *Manual selection* Direct specification of the intensity threshold based on user expertise
  - *Automatic selection* Histogram-based analysis using, where the histogram mean serves as the isovalue.
  - *Interactive selection* Real-time isosurface visualization using vedo’s IsosurfaceBrowser interface with GPU acceleration for immediate feedback
- *Mesh optimization* Following surface extraction:
  - The largest connected component is isolated using to remove spurious surface fragments
  - Mesh complexity is reduced using Quadric Edge Collapse Decimation with a default target reduction factor of 0.005, balancing geometric fidelity with computational efficiency
  - The resulting triangulated surfaces are stored in VTK format

#### Developmental staging through geometric analysis

Limb developmental staging is implemented via a semi-automated approach combining manual landmark placement with algorithmic stage determination. The process utilizes vedo’s SplinePlotter interface where users define anatomical landmarks along the limb contour through point-and-click interaction.

- *Geometric processing* User-defined points undergo plane fitting using least-squares optimization: $\textbf{n} = \text {fit\_plane}(\text {points}).\text {normal}$. A linear transformation matrix is computed to reorient the fitted plane normal to the canonical direction $[0, 0, -1]$: $\textbf{T} = \text {LinearTransform}().\text {reorient}(\textbf{n}, [0, 0, -1], \text {xyplane}=\text {True})$.
  *Stage computation* Point coordinates are transmitted to a dedicated staging service (https://limbstaging.embl.es/api) using JSON formatting of the position of the selected points. The API processes the landmark coordinates and returns the developmental stage classification based on established morphometric criteria.

#### Spatial alignment and registration

Limb alignment to reference models is accomplished through two distinct approaches, each addressing different aspects of morphological variation.

##### Rigid registration (linear)

Rigid alignment performs 6-DOF (degrees of freedom) transformation estimation through interactive mesh alignment. The source mesh is compared against a reference model selected from a database of 42 developmental stages (stages 249–290) stored as VTK mesh files. The transformation estimation employs interactive manipulation tools with real-time visual feedback through a multi-viewport visualization system. The resulting $4 \times 4$ homogeneous transformation matrix encodes rotation, translation, and scaling operations: $$\textbf{T} = \begin{bmatrix} \textbf{R} & \textbf{t} \\ \textbf{0}^T & 1 \end{bmatrix}$$ where $\textbf{R}$ is the $3 \times 3$ rotation matrix and $\textbf{t}$ is the translation vector.

##### Non-rigid registration (non-linear)

 Non-rigid alignment implements Thin Plate Spline (TPS) deformation for more sophisticated shape matching. The vedo’s MorphPlotter interface facilitates interactive landmark correspondence establishment between source and target meshes. The TPS warping function minimizes the bending energy. The resulting non-linear transformation captures local shape variations while maintaining global topology.

### Visualization and analysis tools

The visualization subsystem provides five distinct rendering algorithms, each optimized for specific analytical tasks:

- *Isosurface rendering* Surface visualization with support for single and dual-channel rendering. Multiple isovalues are computed using linear interpolation and rendered with alpha blending for volumetric depth perception. While the rendering system imposes no theoretical constraints on the number of isosurfaces that can be simultaneously displayed, we implemented an arbitrary upper limit of 10 isosurfaces to maintain visualization clarity and prevent visual clutter that could impede data interpretation.
- *Volume ray casting* Direct volume rendering using the vedo’s RayCastPlotter interface, enabling visualization of internal structures through opacity transfer functions and colour mapping schemes.
- *Slab visualization* Interactive thick-slice exploration allowing real-time adjustment of slice thickness and position through user interface controls, facilitating layer-by-layer analysis of gene expression patterns.
- *Orthogonal slicing* Multi-planar reconstruction generating 2D cross-sections at arbitrary orientations for detailed structural examination.
- *Interactive probing* Spline-based sampling tools (probe) enabling quantitative intensity profiling along user-defined paths, with real-time plotting of signal intensity distributions.

All visualization components support transformation application (both linear and non-linear) ensuring that analytical results remain consistent across different coordinate systems and developmental stages. 

## Availability

- Project name: LimbLab
- Project home page: https://limblab.embl.eshttps://limblab.embl.es/
- Operating system(s): Platform independent (tested in Windows)
- Programming language: Python
- Other requirements: Python$\geqslant$3.7
- License: MIT
- Any restrictions to use by non-academics: No

## Additional file

Supplementary Material 1.

## Data Availability

Data used for the figures can be found in BioImage Archive S-BIAD1925 and BioImage Archive S-BIAD1926.
